# Polymeric Nanocomposites of Polyvinyl Alcohol Embedded with ZnO/CuO/Single-Walled Carbon Nanotubes: Optical and Radiation Shielding Investigations

**DOI:** 10.3390/polym17060818

**Published:** 2025-03-20

**Authors:** Sami S. Alharthi, Ali Badawi

**Affiliations:** 1 Department of Physics, College of Science, Taif University, P.O. Box 11099, Taif 21944, Saudi Arabia; s.saeed@tu.edu.sa; 2 Department of Physics, University College of Turabah, Taif University, P.O. Box 11099, Taif 21944, Saudi Arabia

**Keywords:** PVA polymeric nanocomposite, ZnO/CuO/SWCNTs, linear/nonlinear optical, optical bandgap, linear attenuation coefficient, radiation shielding parameters

## Abstract

The optical and radiation shielding of PVA have been enhanced through embedding with ZnO/CuO/SWCNT (ZCS) nanocomposites. ZCS polymeric nanocomposites (PNCs) were prepared with the solution casting method. Scanning electron, optical microscopy and FT-IR procedures were performed to examine the surfaces’ morphology and structures’ modifications. UV–visible measurements were carried out to investigate the linear/nonlinear optical properties. The optical investigations show significant alterations in the optical parameters of PVA due to ZCS embedding. The UV–visible analysis shows that the optical parameters, including the transmittance, energy bandgap, refractive index, dielectric constants and optical conductivity of PVA, are tuned through ZCS embedding. The direct and indirect bandgap of PVA shrank from 5.42 eV and 4.99 eV (neat PVA) to 3.20 eV and 2.26 eV (10 wt.% ZCS PNCs). The nonlinear optical (NLO) constants (first order susceptibility (χ^(1)^), third susceptibility (χ^(3)^) and refractive index (n_2_)) of PVA were improved. Phy-X/PSD software was used to investigate the radiation shielding parameters of all samples. The linear attenuation coefficient (LAC), mean free path (MFP), half value layer (HVL), tenth value layer (TVL) and effective atomic number (Z_eff_) of PVA were enhanced through ZCS embedding. It is found that the mass attenuation coefficient (MAC) of the neat PVA increased from 1.14 cm^2^/g to 7.96 cm^2^/g at 0.015 MeV. The HVL of PVA decreased from 30.2 cm to 20.6 cm, the TVL decreased from 100.3 cm to 68.5 cm and the MFP decreased from 43.6 cm to 29.8 cm upon embedding 10 wt.% of ZCS NCs at 15 MeV. The samples’ exposure buildup factor (EBF) and energy absorption buildup factor (EABF) in the photon energy range from 0.015 MeV to 15 MeV at 0.5 to 40 MFP values. This study proves that ZCS PNCs are advantageous for applications in optical and radiation shielding fields.

## 1. Introduction

Recently, polymeric nanocomposites have played effective roles in different fields, including the optoelectronic, biomedical and entertainment fields [1,2,3,4]. The features of polymeric nanocomposites (PNCs), such as flexibility, their properties’ tuning opportunity, availability and low cost, make them favorable for use in solar cells, optics, sensors and communications [5,6,7,8,9]. PNCs could be produced by embedding a dopant in a polymeric host for such an application. Polyvinyl alcohol (PVA) possesses many outstanding features, such as its water solubility, environmental friendship, degradability and biocompatibility [10,11]. Due to these features, PVA has been used for food packaging, biomedicine, ceramics and fiber production [12]. Moreover, the PVA optical transmittance of the visible–IR spectra makes it suitable for optical window applications. In addition, the capability of film production due to the existence of –OH qualifies it as a unique host of a lot of additives and dopants [6]. The wide bandgap character of PVA (~5.4 eV [6]) provides the opportunity to tune its optical parameters for fabricating plenty of optical devices. For example, Mostafa et al. [13] used PVA as a host matrix for Co_0.2_Zn_0.2_Cd_0.6_Fe_2_O_4_ nanoparticles (NPs) for optical applications. The electrical conductivity of PVA was tuned by Ragab [14] from 4.76 × 10^−8^ S·cm^−1^ to 3.67 × 10^−5^ S·cm^−1^ through Co_3_O_4_ incorporation. Iswarya’s research group showed that PVA embedded with silver NPs played the role of an effective solid electrolyte in electrochemical devices [15]. Dangi et al. [12] used reduced graphene oxide (rGO) to control the optical bandgap of PVA from 4.57 eV to 2.67 eV for optoelectronic device applications. Alharthi’s group [16] proved that CuO/rGO-doped PVA played an effective role in irradiation applications. The radiation shielding performance of polyvinyl chloride was pronouncedly enhanced by Heiba et al. [17] through filling with cobalt/erbium ferrite. Iswarya et al. [15] proved that Ag NPs doped in PVA possessed a high anti-bacterial activity for food packing. Alshammari used SrTiO_3_/CNT NCs to enhance the thermal and optical performance of PVA for optoelectronic application [18]. Alhuthali et al. [19] reported that the PVA/graphene PNCs could be an active candidate in flexible solar cells. Alharshan’s group concluded that MnCl_2_ obviously enhanced the PVA attenuation performance of the gamma rays. Furthermore, Issa et al. [2] reported that BaTiO3 NPs significantly improved the PVA’s mass attenuation coefficient.

The present work aims to reinforce the optical and radiation shielding of PVA by incorporating ZnO/CuO/single-walled carbon nanotube (SWCNT) nanocomposite for optoelectronic and radiation shielding applications. To our knowledge, the ZnO/CuO/SWCNT (ZCS) nanocomposite has not been used as a dopant in PVA before for such an application. The SWCNTs possess many attractive characteristics, such as being lightweight, having biocompatibility and being eco-friendly [20,21]. Moreover, the high mechanical and electrical performance with a large surface area of the SWCNTs enriches all the physical and chemical properties of the host medium. SWCNTs play a positive role in enhancing the fillers’ distribution in the PVA host and improving the homogeneity character of the samples. The ZnO/CuO nanocomposite possesses plenty of favorable features, such as nontoxicity, environment friendliness, low cost and availability. In addition, the optical properties of the ZnO/CuO nanocomposite make it a novel dopant in PVA that qualifies it for a lot of applications in energy technologies, ceramics, optoelectronics and radiation protection [22]. Saravanan et at. [23] reported that the optical properties of the coupled ZnO/CuO nanocomposites are preferable for photocatalytic applications as compared with single semiconductors. Similarly, Safdar’s research group reported that ZnO/CuO nanocomposites play a significant role in different optoelectronic applications [24].

In the current work, different contents of ZnO/CuO/SWCNT nanocomposite embedded in PVA were fabricated using the solution casting technique. The optical parameters of the PNCs were studied based on the UV–visible measurements. The surfaces and bulk morphology were captured using a scanning electron and optical microscopy technique. The structure of all PNCs was examined by Fourier transform infrared (FT-IR) spectroscopy. The radiation shielding parameters were found using Phy-X/PSD software [25]. The outcomes of this study indicate that ZnO/CuO/SWCNT PNCs could play an effective role in various applications with respect to optical and radiation shielding issues.

## 2. Materials and Methods

**Figure 1:**
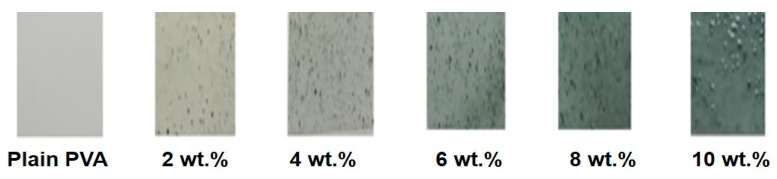
Images of neat and different (2 to 10 wt.%) ZCS PNCs.

The PVA granules (86–89% hydrolyzed; M.W.: 85,000 g/mol.) were provided by Alfa Aesar Co., Ward Hill, MA, USA, The SWCNTs, of 30 μm length, diameter of 2 nm and purity of 99.99%, were supplied by Chengdu organic chemicals Co. (Chengdu, Sichuan, China). The chemicals ZnCl_2_ (purity > 98%), CuCl_2_·2H_2_O (purity > 99%) and NaOH (purity ≥ 95%) were purchased from Sigma-Aldrich Co., St. Louis, MO, USA. Distilled water (DW) was used as a solvent for all previous materials. The chemical bath deposition technique was followed to prepare ZnO/CuO/SWCNT nanocomposite, as shown in [26,27]. Briefly, an ionic solution of Zn^2+^, Cu^2+^ and SWCNTs with weight ratios of 2:1:0.02 was prepared by dissolving and stirring their precursors at 30 °C for 60 min. Next, drops of NaOH solution were added to achieve a pH of 11. After that, a brown-black precipitate was obtained. The obtained precipitate was washed several times with distilled water to remove extra residues and impurities and then dried at 80 °C for 12 h. Finally, the obtained product was calcined for 8 h at 500 °C. The solution casting method was performed to equip the PVA doped with different contents of ZnO/CuO/SWCNTs nanocomposites (ZCS NCs). To prepare a neat PVA, 1.5 g of PVA was dissolved in DW and magnetically stirred at 75 °C for 3 h till a clear solution was achieved. To prepare the ZCS PNCs, a specific amount of the ZCS NCs was added to the previous solution and stirred for an hour at 30 °C. As a result of that, in addition to the neat PVA, five solutions of ZCS PNCs were attained with ZCS contents of 2, 4, 6, 8 and 10 wt.%. After that, the prepared solutions were separately poured into Petri dishes and left for 24 h at 50 °C. The thickness of the obtained films was measured by a micrometer and found to be 0.19 ± 0.01 mm. The films were labeled from a to f, as illustrated in Figure 1.

The structure and chemical bonding of the neat and different PNCs were examined by a Shimadzu Fourier transform infrared (IRAffinity-1S) spectrophotometer (Kyoto, Japan). The bulk morphology of all samples was captured by optical microscopy (Meiji Techno MT5310H Phase, Saitama, Japan) in the reflectance mode with a magnification of 100×. The surface morphology was examined by a scanning electron microscope (SEM). A JASCO UV-Vis-NIR (V670; Easton, MD, USA) spectrophotometer was utilized to collect the optical transmittance (T) and absorbance (A) of the samples in the wavelength range from 200 to 1400 nm. The samples’ optical bandgap (direct and indirect) values were investigated using the Tauc relation [28,29,30,31,32]:

(1) $$\alpha h\nu =G\;{\left(h\nu -E_{g}\right)}^{m}$$

where hν is photon energy, α is the absorption coefficient, E_g_ is the optical bandgap and m is a constant equaling either 0.5 or 2 for the direct and indirect electronic transitions. The Urbach energy (*E_u_*) was determined to investigate the defects and disorder character due to ZCS embedding [7]:

(2) $$\alpha =\alpha_{0}\exp(h\nu /E_{u})$$

where α_0_ is a constant.

The refractive index (n) values of all samples at specific wavelengths (λ) that correspond to their Eg values were calculated using the Dimitrov–Sakka equation, given as [33,34,35]

(3) $$\frac{n^{2}-1}{n^{2}+1}=1-\sqrt{E_{g}/20}\;$$

The dielectric constants (real ε_r_ and imaginary ε_i_) and optical conductivity (σ_opt_.) of the samples were computed using [13]

(4) $$\varepsilon_{r}=n^{2}-K^{2}$$

(5) $$\varepsilon_{i}=2\;nK$$

(6) $$\sigma_{opt.}=\frac{\alpha nC}{4\pi }$$

where C is the light speed and *K* ($=\frac{\alpha \lambda }{4\pi }$) is the extinction coefficient. Furthermore, the nonlinear optical (NLO) behavior of the host PVA polymer was examined as a result of ZCS embedding. Particularly, the 1st order linear optical susceptibility (χ^(1)^), 3rd order NLO susceptibility (χ^(3)^) and NLO refractive index (n_2_) were determined using Tichy et al.’s equations, given as [36,37,38,39]

(7) $$\chi^{\left(1\right)}=\frac{n^{2}-1}{4\pi }$$

(8) $$\chi^{\left(3\right)}=1.7\times 10^{-10}{\left(\chi^{\left(1\right)}\right)}^{4}$$

(9) $$n_{2}=\frac{12\pi }{n}\;\chi^{\left(3\right)}$$

The impact of the ZCS NCs on the radiation shielding performance of the host PVA polymer was explored by Phy-X/PSD software [25]. Principally, the linear attenuation coefficient (LAC) as a function of the photon energy range from 0.015 MeV to 15 MeV of all samples was determined based on the Beer–Lambert law [40]:

(10) $$I=I_{0}e^{-\left(LAC\right)d}$$

Then

(11) $$LAC=\frac{ln\left(\frac{I_{0}}{I}\right)}{d}$$

where *I* and *I*_0_ are the attenuated and initial photon intensity. d is the sample’s thickness. Based on the LAC parameter, the mass attenuation coefficient (MAC), the mean free path (MFB), the half and tenth value layers (HVL, TVL), the effective electron density (Z_eff_), exposure buildup factor (EBF) and the energy absorption buildup factor (EABF) were calculated as [25,41]

(12) $$MAC=\frac{LAC}{\rho }$$

(13) $$HVL=\frac{ln2}{LAC}$$

(14) $$TVL=\frac{ln10}{LAC}$$

(15) $$MFP=\frac{1}{LAC}$$

(16) $$Z_{eff}=\frac{\sum_{i}f_{i}A_{i}\;{\left(MAC\right)}_{i}}{\sum_{j}f_{j}\frac{A_{j}}{Z_{j}}{\left(MAC\right)}_{j}}$$

where ρ is the sample density. *f_i_* and *f_j_* are sample’s molar fractions of the *i*th and *j*th elements, respectively. *A_i_* and *Z_i_* are sample’s atomic weight and number of the *i*th and *j*th elements. Finally, the EBF and EABF parameters were computed in the range from 0.5 to 40 MFP using the geometric progression fitting method [42].

## 3. Results and Discussion

### 3.1. Morphological Investigations

**Figure 2:**
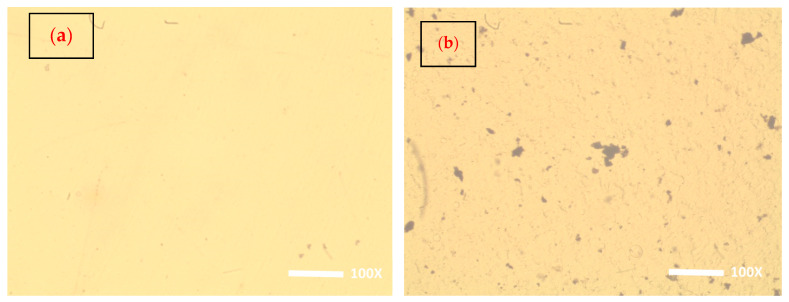

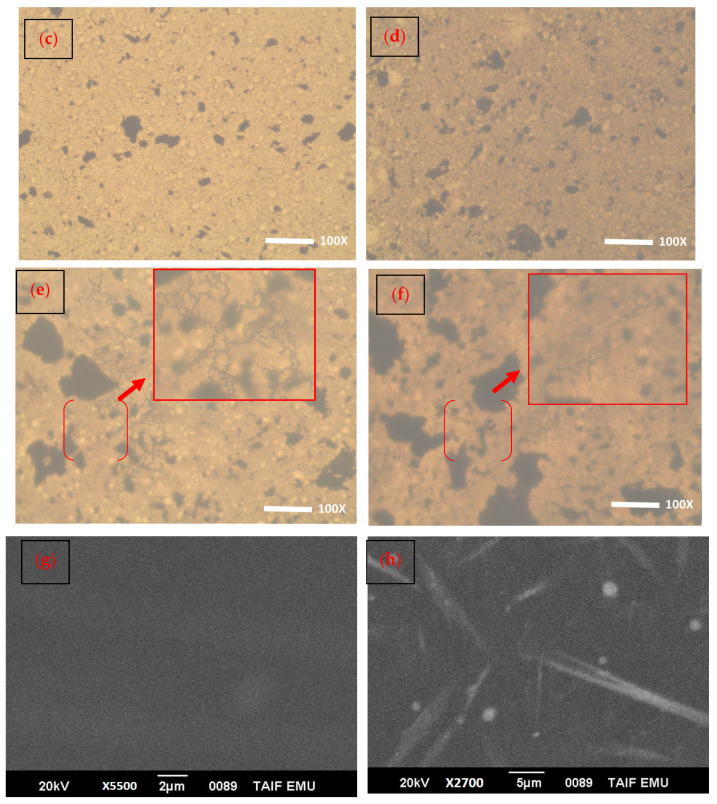
Bulk images of (**a**) neat PVA and (**b**–**f**) ZCS PNCs (2, 4, 6, 8 and 10 wt.%). The insets of Figure (**e**,**f**) show the SWCNTs within the PVA matrix. (**g**,**h**) SEM micrographs of neat PVA and 6 wt.% of ZCS PNCs.

The bulk morphology of the neat PVA host and all PNCs of different ZCS contents (2, 4, 6, 8 and 10 wt.%) was examined using an optical microscope in transmittance mode with a magnification of 100×. This technique presents straightforward information about the samples’ surfaces. The captured surface images of all samples are depicted in Figure 2a–f. It is noted that the surface of the neat PVA is clear without spots and nodes (Figure 2a), while the surface images of the ZCS PNCs indicate the existence of black spots that symbolize the agglomerations of the embedded ZCS NCs. These spots are distributed homogeneously within the host PVA matrix. Moreover, the size of these clusters becomes bigger and denser upon increasing the content of the embedded ZCS NCs. The color of the PNCs becomes darker as the ZCS NCs concentration is increased from 2 wt.% to 10 wt.%. Furthermore, clear branches and nets are detected in the PNCs’ bulks of the large embedded contents of ZCS NCs (8 wt.% and 10 wt.%) (insets of Figure 2e,f). These branches symbolize the SWCNTs that are extended within the host matrix. The optical microscopy examinations ensure the successful integration between the ZCS NCs and the host PVA matrix. Moreover, the scanning electron microscope (SEM) was used to examine the morphology of the sample surfaces, as presented in Figure 2g for neat PVA and Figure 2h for 6 wt.% ZCS PNCs. It is clearly noted that the SEM micrograph of neat PVA is free from any spots, while the SEM micrograph of the ZCS PNCs shows clear spots and quasi rods related to SWCNTs.

### 3.2. FT-IR Analysis

**Figure 3:**
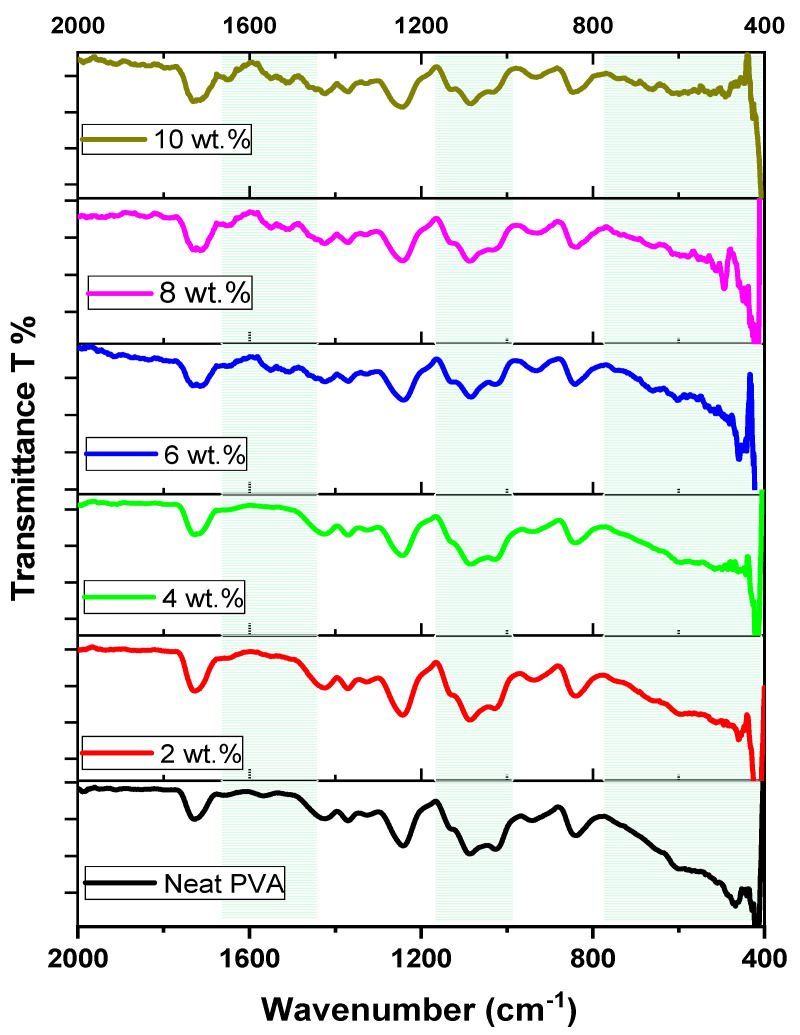
FTIR spectra of neat PVA and different (2 to 10 wt.%) ZCS PNCs. The back-green areas denote to the FT-IR spectra changes.

The FT-IR spectroscopy technique was utilized to explore the impact of the ZCS NC concentration on the structure of the host PVA medium. Figure 3 illustrates the FT-IR transmittance spectra of the neat PVA and different (2 wt.% to 10 wt.%) concentrations of ZCS NCs in the 400 to 2000 cm^−1^ wavenumber range. It is noted that all samples possess the same absorption bands, which correspond to the PVA polymer with clear variations in their intensities and locations. The PVA absorption band that was detected at 1730 cm^−1^ refers to the stretching vibration of C=O bonds [43,44]. The peaks at 1644 cm^−1^ and 1548 cm^−1^ detected in all FT-IR spectra of ZCS PNCs refer to the stretching vibrations of C=O and C=C bonds for SWCNTs [28,45]. The absorption peaks located at 1428 cm^−1^, 1370 cm^−1^ and 1245 cm^−1^ are ascribed to the PVA bending of C-H [28,46], wagging of C-H [46] and stretching of C-C [47] bonds, respectively. The peaks at 1087 cm^−1^, 1020 cm^−1^ and 838 cm^−1^ are assigned to stretching vibrations of PVA’s C-O [48], C-O [49] and C-C [28,48] bonds, respectively. The intensity of these peaks varies from the neat and ZCS-embedded PVA due to the interaction of the NCs with the skeleton of the host PVA matrix. The absorption peak at 650 cm^−1^ is assigned to OH wagging vibration [28]. The variation in the intensity of the broad absorption band detected in the wavenumber range from 400 cm^−1^ to 750 cm^−1^ is attributed to the interaction with Zn-O and Cu-O stretching bonds and OH wagging vibration. These stamped changes noticed in the FT-IR spectra of the ZCS PNCs relative to the neat PVA ensure the successful interactions between the nanofillers and the chains of the PVA host. Similar findings are reported in the literature [50,51].

### 3.3. Optical Analysis

**Figure 4:**
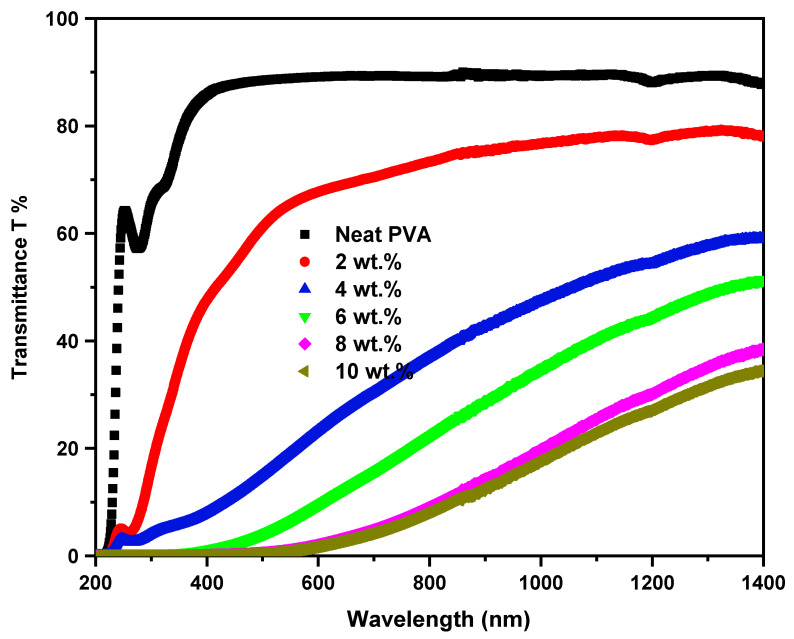
Transmittance spectra of neat PVA and different (2 to 10 wt.%) ZCS PNCs.

The insight variations in the optical performance of the host PVA due to ZCS NCs embedding were studied based on the UV-Vis. measurements. Investigating the optical properties of the ZCS PNCs is essential to recommend their applications in fabricating optical devices. The concentration impact of the ZCS NCs embedding on the optical transmittance (T) of the PVA was examined in the wavelength range from 200 nm to 1400 nm, as depicted in Figure 4. It is obvious that the T of the host PVA reduced upon raising the ZCS NCs contents up to 10 wt.%. Also, clear red-shifts in the cut-off edges to longer wavelengths, from 216 nm (neat PVA) to 560 nm (10 wt.% ZCS PNCs), can be noticed. For example, the 4 wt.% ZCS PNCs could cut off the majority of the UV spectrum (<380 nm), which qualifies it for optical window applications. The rest of the PNCs possess different cut-off edges, which qualify them as filters. The reduction in the transmittance and red-shift observations are attributed to increases in the absorption that reveal a shrinkage of the PVA optical bandgap as a result of ZCS embedding. The incorporation of the ZCS NCs with the PVA host creates localized energy states between the highest occupied molecular orbital (HOMO) and lowest unoccupied molecular orbital (LUMO) of PVA. This behavior leads to a decrease in the optical bandgap and, hence, the cut-off edge is red-shifted. The capability of tailoring the cut-off edge of PVA optical transmittance nominates the ZCS PNCs for applications in optical windows and filters. Furthermore, the absorption edges found at 279 nm and 330 nm in the T spectrum of the neat PVA are assigned to π→π^*^ and n→π^*^ bands transitions [2].

**Figure 5:**
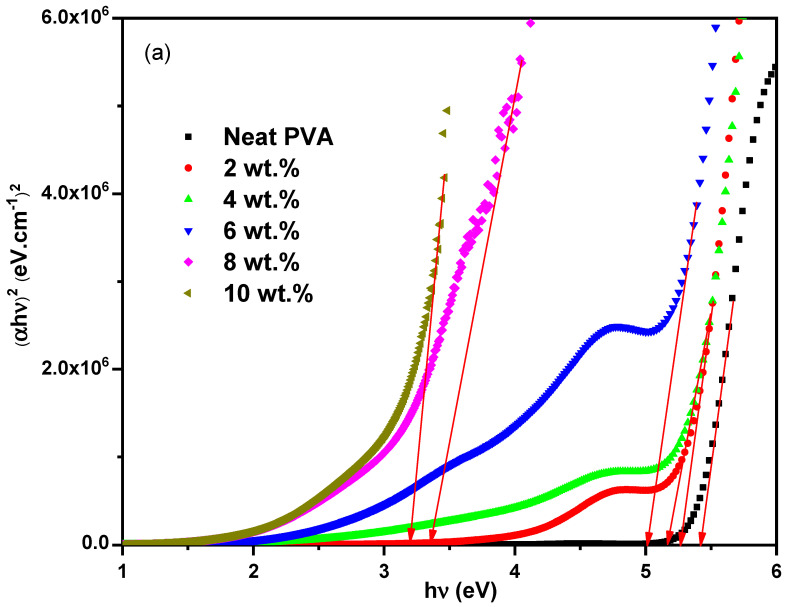

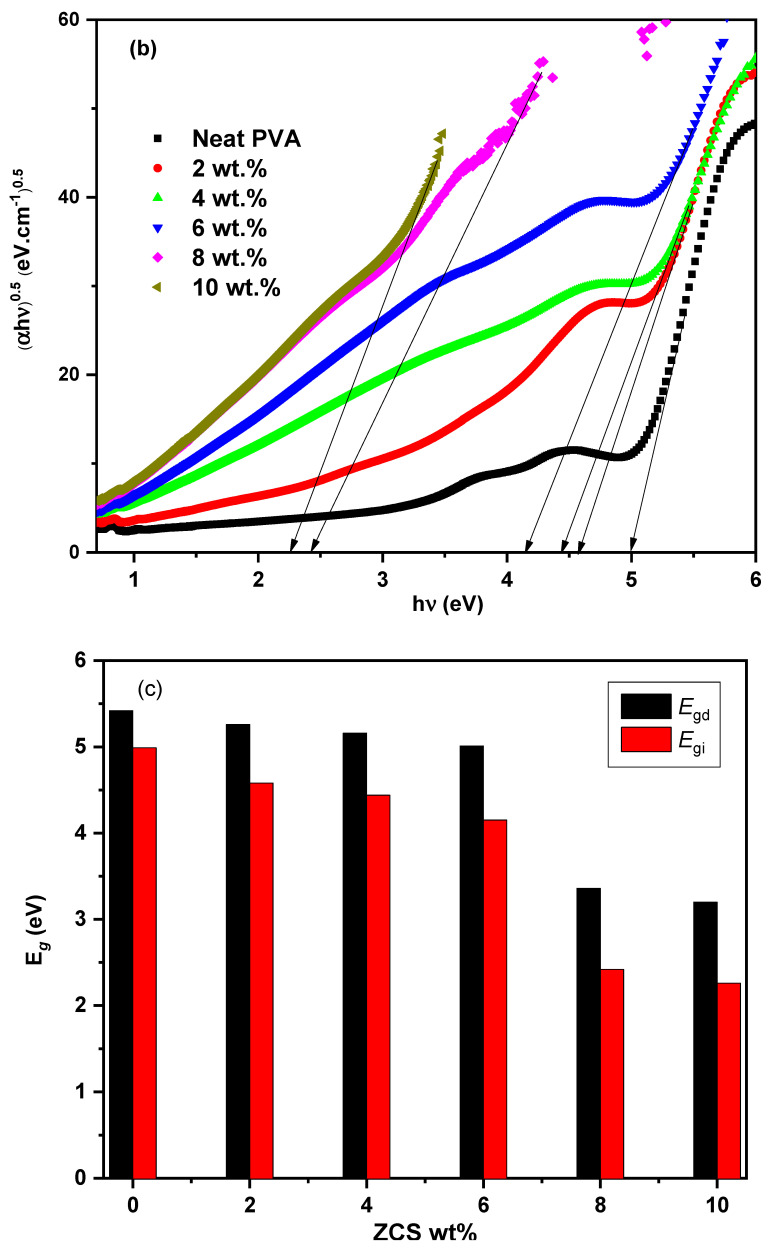
Tauc’s plots; (**a**) direct and (**b**) indirect transitions of neat PVA and different (2 to 10 wt.%) ZCS PNCs, (**c**) energy bandgap dependence on the ZCS contents.

The impact of the ZCS embedding on the PVA optical bandgap (E_g_) was explored using Tauc’s method (Equation (1)). The relation between (*αhν*)^2^ and (*αhν*)^0.5^ and *hν* for the neat PVA and different ZCS PNCs is presented in Figure 5a,b to investigate the direct and indirect E_g_ values, respectively. The intersection of the extended linear parts of the plotted curves to *hν* = 0 equals the E_g_ values. It is found that both direct and indirect E_g_ values of PVA decreased from 5.42 eV and 4.99 eV (neat PVA) to 3.20 eV and 2.26 eV (10 wt.% ZCS PNCs), as presented in Figure 5c. These results are compatible with the optical transmittance findings. The reduction in the PVA E_g_ value is understood in terms of the localized energy levels created between the highest occupied molecular orbital and the lowest unoccupied molecular orbital of the host PVA due to ZCS embedding [5,7]. Additionally, the ZCS embedding results an increment in the vacancies and defects in the PVA host [52]. Tuning the optical bandgap of PVA through ZCS embedding is considered a novel result of the current study, which nominates the ZCS PNCs for a lot of applications in optical devices, such as LEDs, photovoltaics and solar cells. The same observations have been reported in previous works [11,13,53].

**Figure 6:**
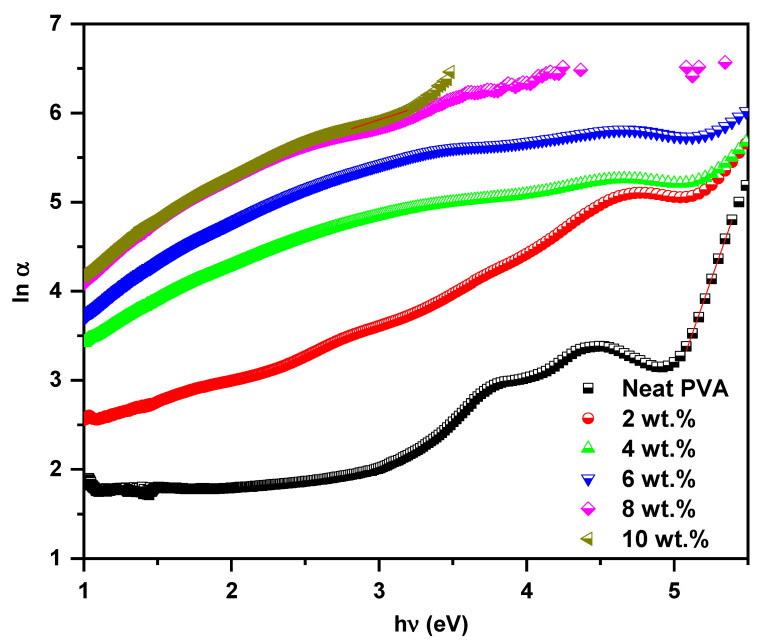
Urbach energy determination of neat PVA and different (2 to 10 wt.%) ZCS PNCs.

The interpretation of the optical bandgap reduction in the PVA host as a result of ZCS embedding is verified on the basis of Urbach energy investigation (Equation (2)). The relation between the logarithm of the absorption coefficient (ln*α*) and photon energy (*hν*) of the neat PVA and all ZCS PNCs is presented in Figure 6. The Urbach energy (*E_u_*) value equals the slope’s inverse of the linear and fits just below the optical bandgap values. It is found that the *E_u_* of the neat PVA is 0.21 eV, and it rises up to 1.85 eV for the 10 wt.% ZCS PNCs. This result indicates that the disorder feature and vacancies density of the PVA host increased considerably due to ZCS embedding [54,55]. The increment in the vacancies density and localized energy states of PVA due to ZCS embedding led to the optical bandgap reduction and, hence, improved the absorption.

**Figure 7:**
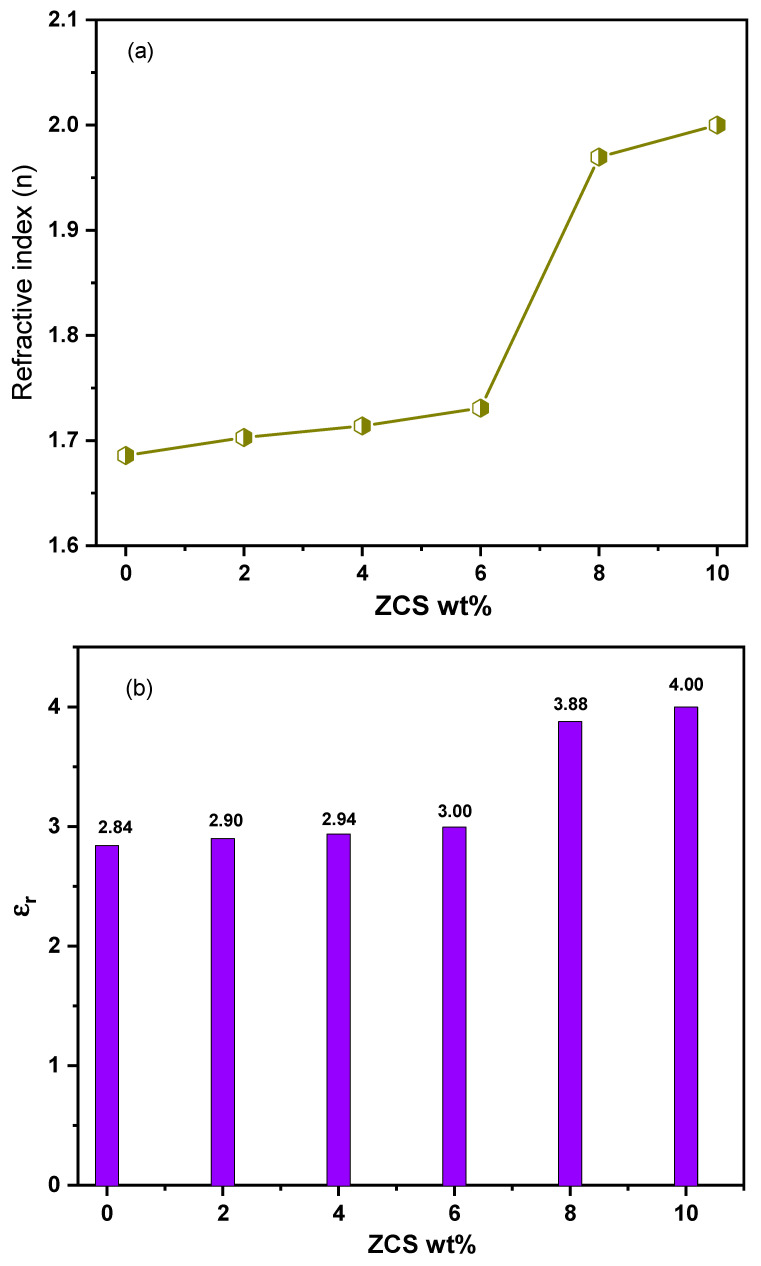

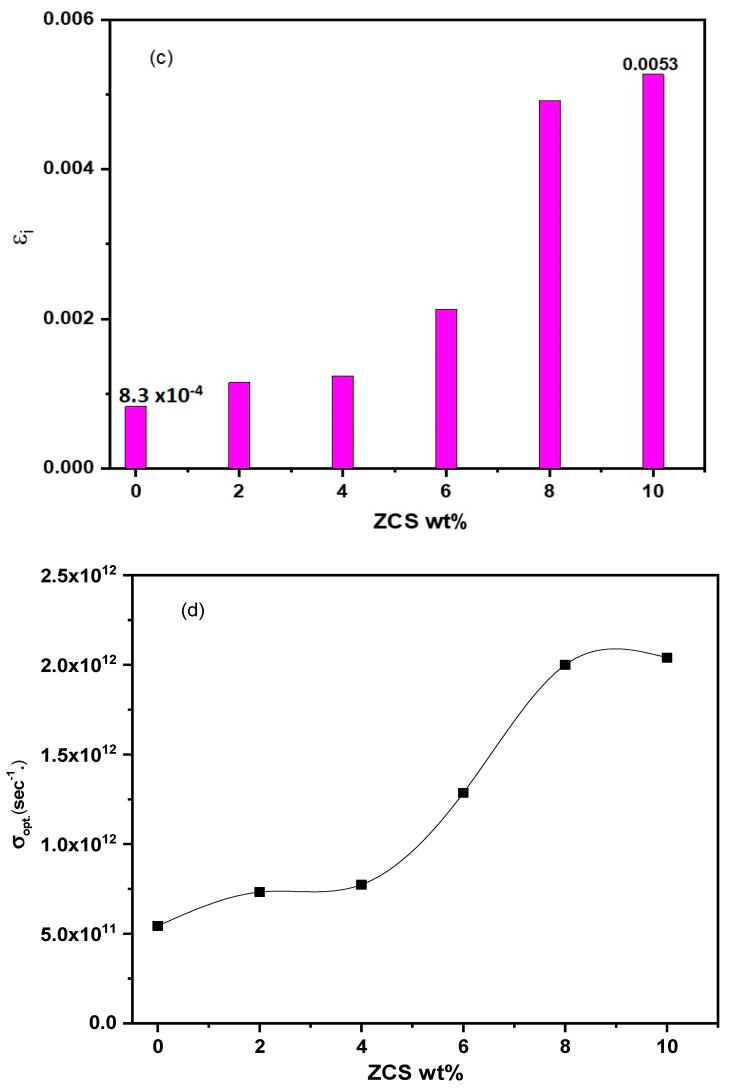
(**a**) Refractive index, (**b**) real, (**c**) imaginary dielectric constants and (**d**) optical conductivity of neat PVA and different (2 to 10 wt.%) ZCS PNCs.

The refractive index is a crucial parameter in recommending the validity of the ZCS PNCs for applications in optical devices. The refractive index of an optical material presents information about its performance due to electromagnetic wave propagation. The values of the refractive index (n) of the neat PVA and all ZCS PNCs at a photon energy equal to their optical bandgap values were computed by the Dimitrov–Sakka equation (Equation (3)) and are illustrated in Figure 7a. It is noticeable that the n value of the neat PVA increased gradually from 1.69 to 1.73 upon raising the concentration of ZCS NCs from 0 to 6%. In contrast, it increased rapidly to 1.97 and 2.0 as the ZCS content rose to 8% and 10%. These findings are discussed in terms of the reflectance increase as a result of the increment in the vacancies and defects due to ZCS embedding. Also, the increase in ZCS contents led to a growth in the polarization and refractive index [56]. Moreover, the increment in ZCS embedding increases the packing density, hence enhancing the refractive index [57]. The same trend related to Co_0.2_Zn_0.2_Cd_0.6_Fe_2_O_4_ doped in PVA was reported by Mostafa et al. [13]. The refractive index enhancement of PVA through ZCS embedding qualifies it for microlens components, waveguides, reflecting and anti-reflecting surfaces, image sensors and optical communications [13,58]. Moreover, the impact of ZCS concentration on the dielectric constants (real ε_r_ and imaginary ε_i_) of PVA has been investigated. The ε_r_ and ε_i_ constants of all samples were calculated using Equations (4) and (5) and are illustrated in Figure 7b and c, respectively. Obviously, the ε_r_ and ε_i_ of PVA are enhanced from 2.84 and 0.00083 to 4 and 0.0053 as a result of ZCS embedding. The enhancement in the ε_r_ and ε_i_ constants is attributed to the improvement in the absorption and polarization, respectively. Similar observations are reported in Refs. [11,59,60]. These ε_r_ and ε_i_ findings are preferable in fabricating supercapacitors and energy storage devices. Furthermore, the optical conductivity (σ_opt_.) is another optical parameter that deals with the optical response to the incident photons and the production of the charge carriers. σ_opt_. values of the neat PVA and all ZCS PNCs were calculated by Equation (6) and are depicted in Figure 7d. According to that, the σ_opt_. of PVA was enhanced significantly upon the increments in ZCS contents up to 10%. The σ_opt_. improvement is mainly attributed to the absorption enhancement and, hence, an increase in the charge carrier production. Moreover, the increment in ZCS contents facilitates the charge carriers’ transition and hopping [13].

**Figure 8:**
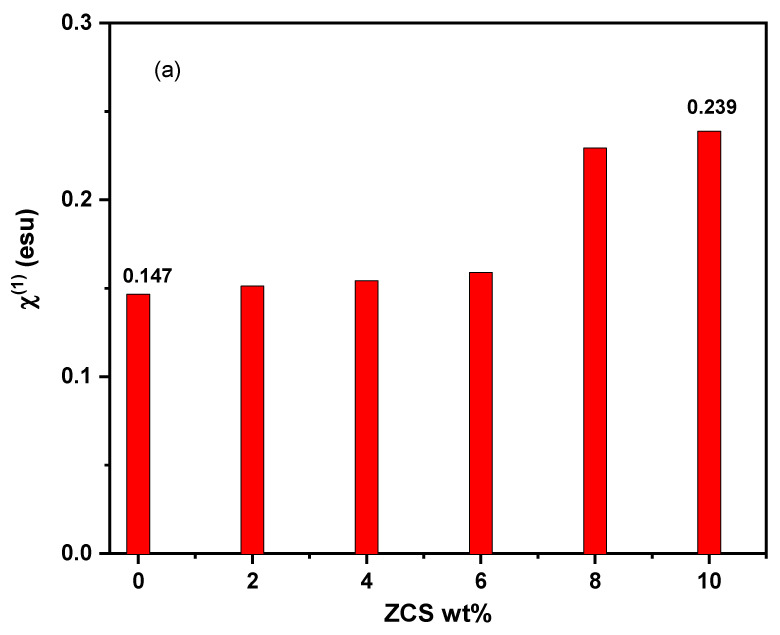

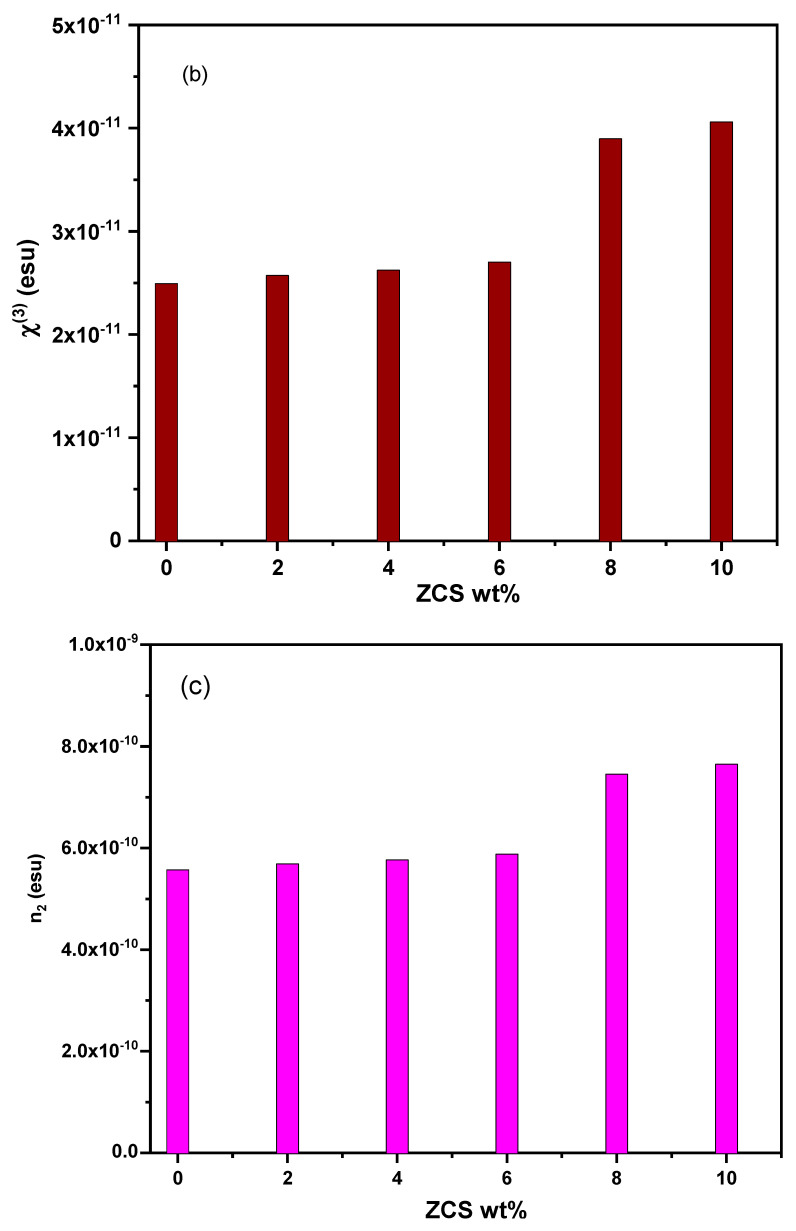
(**a**) χ^(1)^, (**b**) χ^(3)^ and (**c**) n_2_ of neat PVA and different (2 to 10 wt.%) ZCS PNCs.

The nonlinear optical (NLO) performance of neat PVA and different ZCS PNCs has been explored to establish their appropriateness in NLO requests. The NLO applications include 3D optical data storage, frequency converters, optical limiting, lasing switching and telecommunications [61,62,63,64,65]. The NLO response deals with the nonlinear polarization that appears as a result of electromagnetic wave propagation. Mainly, the first linear optical susceptibility (χ^(1)^), third NLO susceptibility (χ^(3)^) and NLO refractive index (n_2_) were computed using Tichy et al.’s equations (Equations (7)–(9)) and are displayed in Figure 8a, b and c, respectively. Noticeably, all NLO parameters (χ^(1)^, χ^(3)^ and n_2_) of the PVA were enhanced considerably through ZCS embedding. Particularly, χ^(1)^ increased from 0.147 esu (neat PVA) to 0.239 esu (10% ZCS PNCs), i.e., about 63% enhancement. The χ^(3)^ of PVA increased from 2.49 × 10^−11^ esu to 4.06 × 10^−11^ esu, and the n_2_ rose from 5.57 × 10^−10^ esu to 7.65 × 10^−10^ esu due to 10 wt.% ZCS embedding. This valuable enhancement in the NLO parameters is attributed to the reinforcement of PVA’s covalence and ionic chemical bonds by ZCS embedding [62]. These results nominate the fitness of ZCS PNCs for a lot of NLO applications [61,66,67]. Similarly, Zyoud et al. [68] reported that ZnO boosted the NLO parameters of the PVA/PVP blend for optical limiting applications.

### 3.4. Radiation Shielding Investigations

**Figure 9:**
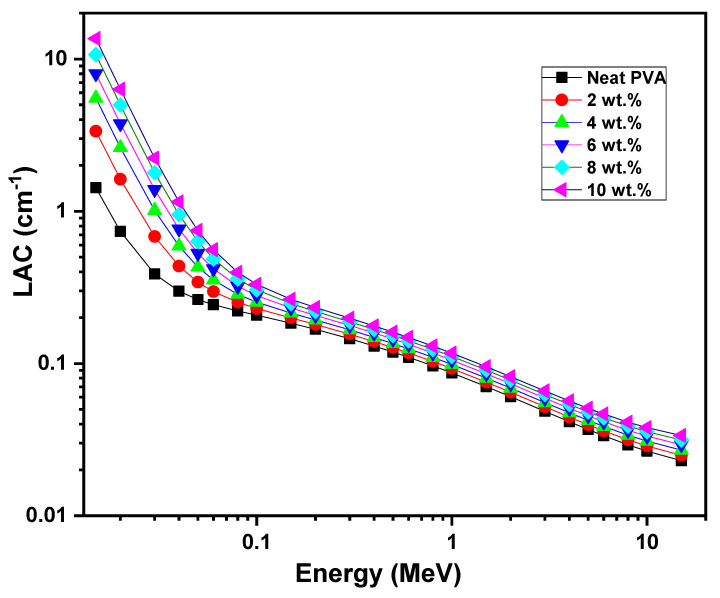
LAC of neat PVA and different (2 to 10 wt.%) ZCS PNCs.

The samples’ performance in the radiation shielding application was explored using Phy-X/PSD software. Many previous works reported in the literature showed the compatibility between the experimental radiation shielding findings and those obtained using Phy-X/PSD software [25,34,69,70]. The linear attenuation coefficient (LAC) values of the neat PVA and different contents (2, 4, 6, 8 and 10 wt.%) of ZCS embedded in PVA at a wide range of photon energies (0.015 MeV to 15 MeV) were calculated (Equation (11)) and are illustrated in Figure 9. It is clear that the LAC of each sample decreased upon increasing the photon energy up to 15 MeV. Also, an increment in the LAC of the PVA can be noticed as the ZCS concentration increases. For example, the LAC of PVA was enhanced by one order of magnitude as a result of 10 wt.% embedding of ZCS NCs at a photon energy of 0.015 MeV. The increment in LAC due to ZCS embedding is mainly attributed to the photoelectric effect and density increase [71,72]. More ZCS embedding in PVA leads to more interaction between the photons and the medium atoms and, hence, enhances the γ-radiation protection characteristics [71,73].

**Figure 10:**
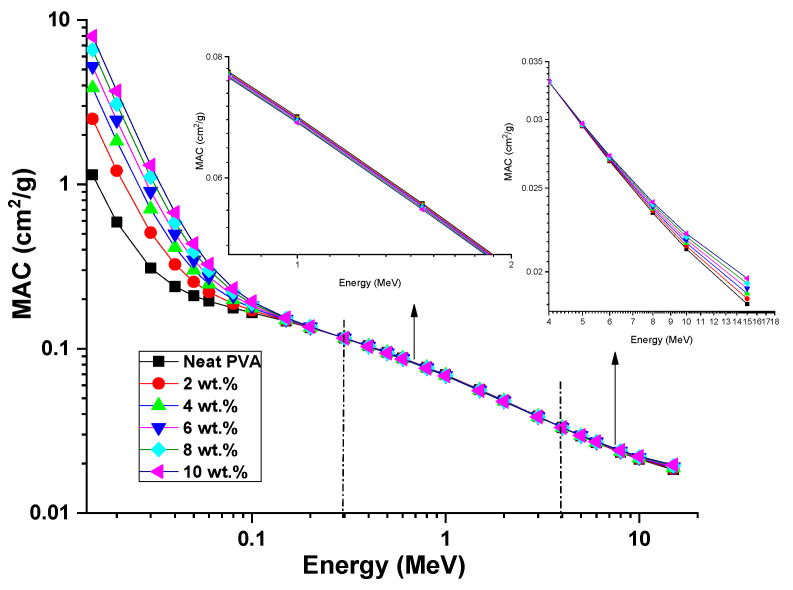
MAC of neat PVA and different (2 to 10 wt.%) ZCS PNCs. Insets show MAC in photon energy range from 0.3–4 MeV and 5–15 MeV.

The values of the mass attenuation coefficient (MAC) as a radiation shielding factor of the neat PVA and different ZCS PNCs were calculated (Equation (12)) over the photon energy range from 0.015 MeV to 15 MeV, as depicted in Figure 10. It can be seen that the MAC behavior of all samples follows echoes of the LAC over the photon energy range. For all samples, the MAC decreased upon increasing the photon energy. The impact of ZCS embedding could be divided into three distinct regions. The first region ranged from 0.015 MeV to 0.2 MeV; the MAC of PVA increased obviously upon increasing the embedding content of ZCS NCs. In an instant, the MAC of PVA at 0.015 MeV increased from 1.14 cm^2^/g to 7.96 cm^2^/g due to 10 wt.% of ZCS embedding. This phenomenon is attributed to the photoelectric effect. The second region ranged from 0.3 MeV to 4 MeV; the MAC of PVA had a tiny decrease as the ZCS content increased to 10 wt.%, as shown in the inset of Figure 10. For example, the MAC at 0.8 MeV decreased from 0.07718 cm^2^/g to 0.07613 cm^2^/g (10% ZCS embedding). This trend is understood in terms of the Compton scattering interaction [34]. However, the third region ranged from 5 MeV to 15 MeV; the MAC of PVA increased slightly again through the ZCS content increasing, as also clarified in the inset of Figure 10. For example, the MAC at 15 MeV increased from 0.01836 cm^2^/g to 0.01966 cm^2^/g as a result of 10 wt.% of ZCS doping. This tendency is attributed to the pair production [34]. Heiba et al. [17] reported compatible observations related to nano CoFe_1.75_Er_0.25_O_4_ loaded in PVC polymer. Our observations in this study nominate the effective role of ZCS PNCs for γ-radiation shielding and dosimetry applications.

**Figure 11:**
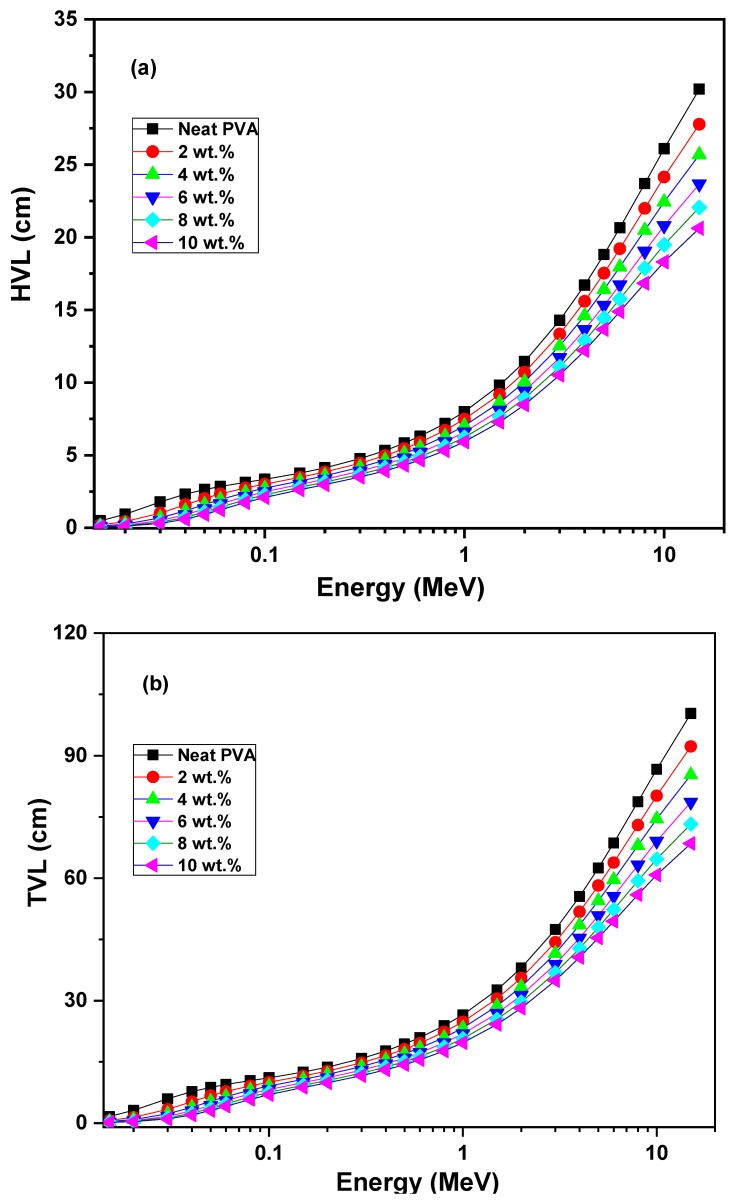

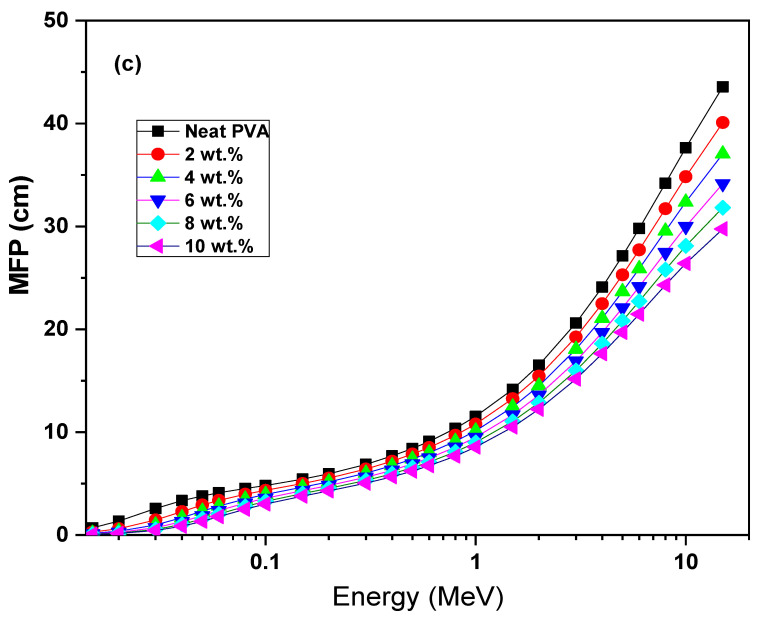
(**a**) HVL, (**b**) TVL and (**c**) MFP of neat PVA and different (2 to 10 wt.%) ZCS PNCs.

Furthermore, the radiation shielding parameters of the half value layer (HVL), tenth value layer (TVL) and mean free path (MFP) related to the neat PVA and ZCS PNCs were also computed (Equations (13)–(15)) over the photon energy 0.015–15 MeV range and are presented in Figure 11a to c, respectively. The HVL factor represents the sample’s thickness where the γ-radiation intensity is reduced to the half value, whereas the TVL is the thickness where the intensity is reduced to the tenth value. The MFP is the mean thickness at which the γ-radiation travelled before attenuation. Noticeably, the samples’ HVL, TVL and MFP perform opposite trends, noted for their LAC and MAC. In other words, HVL, TVL and MFP values increased upon increasing the photon energy. The increment in the ZCS content embedded in the host PVA led to reductions in its HVL, TVL and MFP values. As an example, at 15 MeV, the HVL of PVA decreased from 30.2 cm to 20.6 cm, the TVL decreased from 100.3 cm to 68.5 cm and the MFP decreased from 43.6 cm to 29.8 cm upon embedding 10 wt.% of ZCS NCs. These trends are discussed in terms of the reduction in the γ-ray interaction cross-section phenomenon and LAC values [34]. Increasing the photon energy leads to an increase in the penetrated thickness of the samples. The valuable findings of the HVL, TVL and MFP reveal the validity of ZCS PNCs in applications in gamma radiation shielding. The same observations connected to BiVO_4_ filled in PVC polymer are reported by Kassem et al. [74].

**Figure 12:**
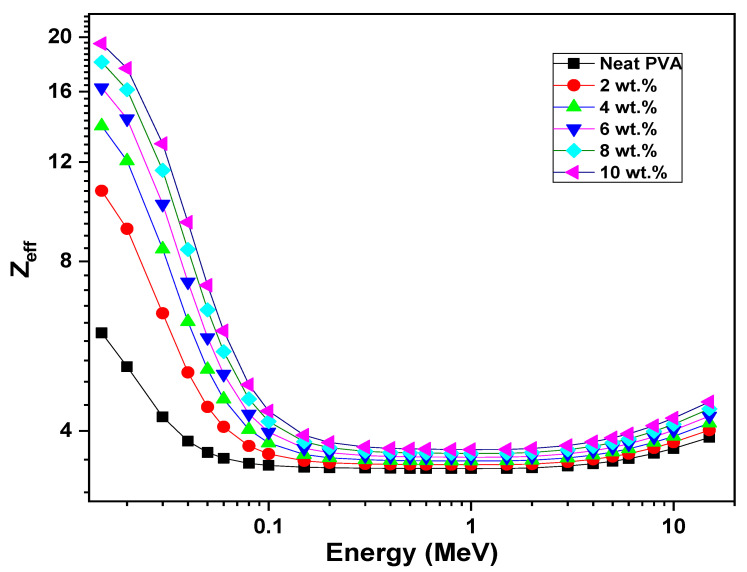
*Z*_eff_ of neat PVA and different (2 to 10 wt.%) ZCS PNCs.

Moreover, the impact of ZCS content and incident photon energy ranging from 0.015 to 15 MeV on the effective atomic number (*Z*_eff_) parameter of PVA have been examined (Equation (16)) and are displayed in Figure 12. The *Z*_eff_ factor represents the effective atomic number at the attenuated photon energy [75]. It is noted that the *Z*_eff_ decreased deeply with increases in the photon energy from 0.015 MeV to 0.1 MeV, then it performed steadily from 0.2 MeV to 3 MeV and increased slowly from 4 MeV to 15 MeV. Furthermore, at any photon energy, the *Z*_eff_ value increased upon the increment in the ZCS content up to 10 wt.%. For example, the *Z*_eff_ of PVA at 0.015 MeV increased from 6 to 19.5 due to 10 wt.% of ZCS embedding. This increment in *Z*_eff_ value is attributed to the photoelectric effect. In addition, increasing the ZCS content leads to an increment in the backing density and, hence, the *Z*_eff_ parameter. Similar trends are reported in previous works [34,74].

**Figure 13:**
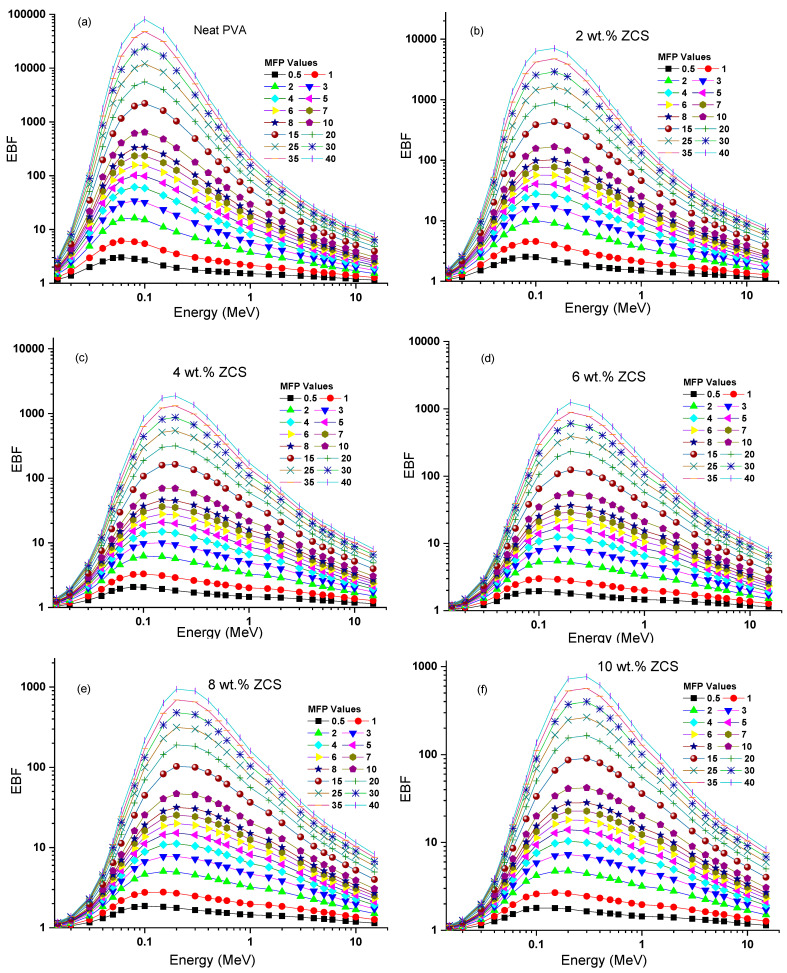
EBF of (**a**) neat PVA and (**b**–**f**) different (2 to 10 wt.%) ZCS PNCs.

**Figure 14:**
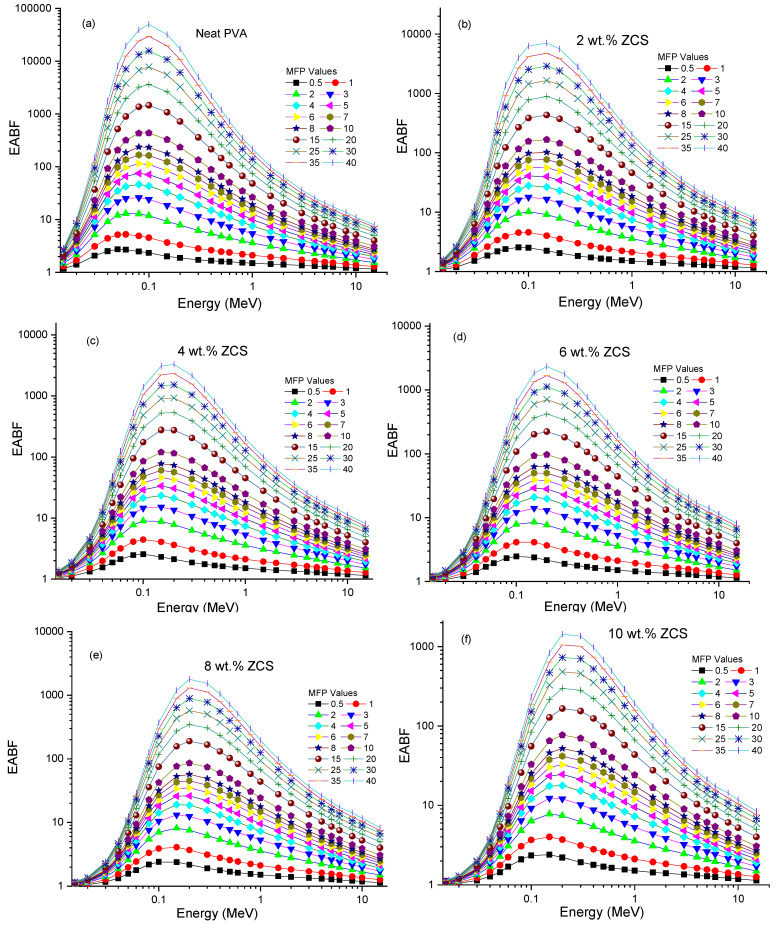
EABF of (**a**) neat PVA and (**b**–**f**) different (2 to 10 wt.%) ZCS PNCs.

Furthermore, the secondary ionizing radiation could be addressed by investigating the buildup factors of the prepared samples [76]. The buildup is divided into factors: exposure buildup factor (EBF) and energy absorption buildup factor (EABF). The EBF and EABF of the neat PVA and different contents of ZCS PNCs versus photon energy (0.015 MeV to 15 MeV) were computed by the geometric progression fitting method at different MFP values (0.5 to 40) and are presented in Figure 13a–f and Figure 14a–f, respectively. Noticeably, as the photon energy increased, each EBF and EABF of a sample increased, reached a peak and then decreased. The maxima points of the EBF and EABF of each sample decreased upon increasing the ZCS content at any specific MFP value. This behavior indicates an enhancement in the radiation shielding feature of PVA through ZCS embedding. The performance of each EBF and EABF comprised three discrete regions of photon energy: low, medium and high. These zones are sequentially connected to the photoelectric effect, Compton effect and pair production [77]. The low-energy regions possess the highest buildup factors, indicating the importance of the photoelectric effect and gamma-ray absorption. The same trends are reported in ref. [78]. The samples’ EBF and EABF depend mainly on their composition, energy range and penetration depth. The EBF and EABF increments indicate the deep penetration of the sample. The findings enclosed in this study prove that the EBF and EABF performance of PVA is significantly affected by embedding with ZCS NCs, nominating it for applications in gamma radiation shielding.

## 4. Conclusions

Neat PVA and different contents (2, 4, 6, 8 and 10 wt.%) of ZnO/CuO/SWCNT nanocomposite (ZCS NC) embedded in PVA were prepared using the solution casting method. The scanning electron and optical microscopy investigations confirm the uniform distribution of ZCS within the PVA medium. The FT-IR investigations indicate a clear modification in the structure and chemical bonding of PVA due to ZCS embedding. The UV–visible analysis shows that the optical parameters, including the transmittance, energy bandgap, refractive index, dielectric constants and optical conductivity of PVA, are tuned through ZCS embedding. A clear red-shift in the transmittance cut-off edges to longer wavelengths, from 216 nm (neat PVA) to 560 nm (10 wt.% ZCS PNCs), was noticed. The direct and indirect bandgaps of PVA shrank from 5.42 eV and 4.99 eV (neat PVA) to 3.20 eV and 2.26 eV (10 wt.% ZCS PNCs). The Urbach energy increased from 0.21 eV to 1.85 eV due to ZCS embedding. The refractive index of PVA was enhanced from 1.69 to 2.0, and the real/imaginary dielectric constants increased from 2.84 and 0.00083 to 4 and 0.0053. The nonlinear optical (NLO) constants (first order susceptibility (χ^(1)^), third susceptibility (χ^(3)^) and refractive index (n_2_)) of PVA were significantly enhanced. The NLO χ^(3)^ of PVA increased from 2.49 × 10^−11^ esu to 4.06 × 10^−11^ esu, and the n_2_ rose from 5.57 × 10^−10^ esu to 7.65 × 10^−10^ esu due to 10 wt.% ZCS embedding. Phy-X/PSD software was used to explore the samples’ radiation shielding parameters. The linear attenuation coefficient (LAC), mean free path (MFP), half value layer (HVL), tenth value layer (TVL) and effective atomic number (*Z*_eff_) of PVA were enhanced through ZCS embedding. The LAC of PVA increased by one order of magnitude. The MAC at 0.015 MeV increased from 1.14 cm^2^/g to 7.96 cm^2^/g due to 10 wt.% of ZCS embedding. At 15 MeV photon energy, the HVL of PVA decreased from 30.2 cm to 20.6 cm, the TVL decreased from 100.3 cm to 68.5 cm and the MFP decreased from 43.6 cm to 29.8 cm upon embedding 10 wt.% of ZCS NCs. The samples’ exposure buildup factor (EBF) and energy absorption buildup factor (EABF) in the photon energy ranged from 0.015 MeV to 15 MeV at 0.5 to 40 MFP values. This study proves that ZCS PNCs are advantageous for applications in optical and radiation shielding applications.

## Data Availability

The original contributions presented in this study are included in the article. Further inquiries can be directed to the corresponding author.
